# Anti-inflammatory and anti-neuropathic effects of a novel quinic acid derivative from Acanthus syriacus 

**Published:** 2019

**Authors:** Karim M. Raafat

**Affiliations:** *Department of Pharmaceutical Sciences, Faculty of Pharmacy, Beirut Arab University, 115020 Beirut, Lebanon*

**Keywords:** Novel quinic acid derivative, Acanthus syriacus, Anti-inflammatory, Antinociceptive effects, Kromeic acid

## Abstract

**Objective:**

*Acanthus syriacus* (AS) is one of the valuable herbal plants with immunomodulatory potentials. The aim of this study is to assemble a phytochemical investigation of *A. syriacus* exploring its anti-inflammatory and antinociceptive properties, identification of its most active compound(s) and elucidating their structure and determining their mechanisms of action.

**Materials and Methods:**

Bio-guided fractionation and isolation-schemes were used utilizing RP-HPLC, CC, ^1^H- and ^13^C-NMR, and biological-models were used to evaluate their effects against inflammation and neuropathic-pain (NP).

**Results:**

The outcomes showed that the most active fraction (FKCA) of AS was identified. Two of the three components of FKCA were identified by chromatographic-methods, while the third compound was isolated, its structure was elucidated and its was named Kromeic acid (KRA); FKCA contained Ferulic acid (27.5%), kromeic acid (48.1%), and chlorogenic acid (24.4%). AS, FKCA and KRA showed significant (p˂0.05) anti-inflammatory and antinociceptive potentials in the management of allodynia and thermal-hyperalgesia in NP. AS and FCKA showed comparatively equipotent antinociceptive-effects. FKCA showed higher antinociceptive effects than KRA suggesting additive-effects among FKCA components. The anti-inflammatory, insulin secretagogue, oxidative-stress reducing, and protective effects against NO-induced neuronal-toxicity might be amongst the possible mechanisms of tested compounds to alleviate NP.

**Conclusion:**

Here, we report the isolation and structure elucidation of a novel quinic-acid derivative, KRA. *A. syriacus,* FKCA, and KRA might be used as a novel complementary approach to ameliorate a variety of painful-syndromes.

## Introduction

The *Acanthus* genus belonging to the Acanthaceae family which is a large plant family consisting of 250 genera and 2700 species distributed widely across the Mediterranean and tropical regions of the world (Capanlar et al., 2010 ▶). The majority of the *Acanthus *species have been used in Asian traditional medicine for amelioration of various ailments. Various *Acanthus *species have shown anti-hepatotoxic, antioxidant, antimicrobial, antitumor, antiviral, anti-inflammatory, analgesic, and anti-fertility activities (Asongalem et al., 2004 ▶; Babu et al., 2001,2002 ▶; Bravo et al., 2004 ▶; Capanlar et al., 2010 ▶). The major constituents of various *Acanthus *species were determined to be alkaloids, flavonoids, glycosides, saponins, and lignans (Bravo et al., 2004 ▶; Capanlar et al., 2010 ▶). In Lebanon, *Acanthus syriacus* is one of the endemic species that has been used as a folk medicine for its immunomodulatory properties and against various neurological disorders (Baydoun et al., 2015 ▶). 

The carrageenan *in-vivo* experiments are widely accepted-models for assessment of the anti-inflammatory effects of compounds and are principally used for the evaluation of the acute-anti-inflammatory potentials of natural or synthetic compounds (Willoughby and DiRosa, 1972 ▶).

Neuropathic pain (NP) is amongst the most difficult pain types to treat after being provoked as a complication of many ailments including diabetes (Ziegler, 2008 ▶). High blood glucose level was shown to provoke allodynia and hyperalgesia in response to chemical or thermal nociceptive provocation (Pabbidi et al., 2008). The NP pathogenesis is multifactorial and the involved mechanisms are not fully understood (Taliyan and Sharma, 2012 ▶). Hyperglycemia also causes reactive-oxygen species (ROS) over-production and innate antioxidant defenses aggravation, increasing the oxidative stress which is an NP fundamental mechanism (Ozkul et al., 2010 ▶). Hyperglycemia provoked oxidative stress and advanced glycation end-product generation leading to stimulation of pro-inflammatory cytokines (CKs) (Cameron and Cotter, 2008). Pro-inflammatory CKs provoke nitric oxide (NO) synthase expression and elevate NO production (Yu et al., 2009; Taliyan and Sharma, 2012 ▶). Moreover, NO is involved in allodynia and hyperalgesia causing the NP (Joharchi and Jorjani, 2007; Taliyan and Sharma, 2012). 

Currently, different classes of non-steroidal anti-inflammatory drugs, opioids, antidepressants, and anticonvulsants are used for amelioration of NP; nevertheless, the limited pain relief is achieved due to their partial-efficiency and recorded toxicities (Ziegler, 2010). Thus, there is an increasing need to discover more efficient and safer drugs for the management of NP. Alternative medicines have gained a reputation in the management of NP, and many indigenous medicinal plants were found to be effective in ameliorating NP (Comelli et al., 2009 ▶; Raafat et al., 2017b ▶; Raafat and Hdaib, 2017 ▶; Taliyan and Sharma, 2012 ▶). Moreover, there is an increasing necessity for deeper investigation of these complementary medicines to understand their chemical compositions and identify their potential compounds and their mechanisms of actions to be used for NP amelioration.

*A. syriacus* is a promising herbal remedy. However, to date, there are no deep phytochemical or biological reports about *A. syriacus*.

Therefore, the aim of the present study is to assemble a phytochemical investigation of *A. syriacus* exploring its anti-inflammatory and antinociceptive properties, identification of its most active compound(s), elucidating active compounds structure and discovering their possible mechanisms of action. 

## Materials and Methods

Standards and solvents were commercially obtained from Merck-Sigma-Aldrich (Germany). 

**Plant material**

The aerial parts of *A. syriacus *were collected from Yahchouch, Kesrwan**, **Mount Lebanon (N 34° 04` 09`` E 35° 44` 20``, Lat: 34.0692911 Lng: 35.7387591), during the flowering stage in the middle of May 2016. The specimen was authenticated with a reference sample and a specimen was deposited in the faculty herbarium with the voucher (No. FP-16-41).

**Extraction**

The aerial parts of *A. syriacus* were dried in shade and size-reduced by G. Ming Mill (China) to form a powder. The dried powder was defatted by hexane and then, sonicated twice using 80% ethanol for 6hr at room temperature. Then, the extract was dried under vacuum at 40°C by Buchi rotary-evaporator (Germany) and then lyophilized utilizing Edwards freeze drier (Germany). The extract was kept frozen at-40°C until further utilization.

**HPLC-PDA analysis:** ***A. syriacus*** **whole extract standardization**

The *A. syriacus* whole ethanolic extract was analyzed utilizing MultoHigh 100 RP18-5µ (Germany) as a reversed phase-high performance liquid chromatography (RP-HPLC) column at 40 °C. The developer was comprised of gradient-elution of (A) Milli-Q distilled water (formic acid 0.1%) and (B) methanol: 0min 90%A; 5 min 72%A; 9 min 55%A; and 14 min 20%A;5μL injection-volume, and flow rate was 1mL/min. The detection wavelength range was 200–600nmfocusing on 254nm. 

***A. syriacus*** **Bio-guided fractionation and isolation**

**Figure 1:**
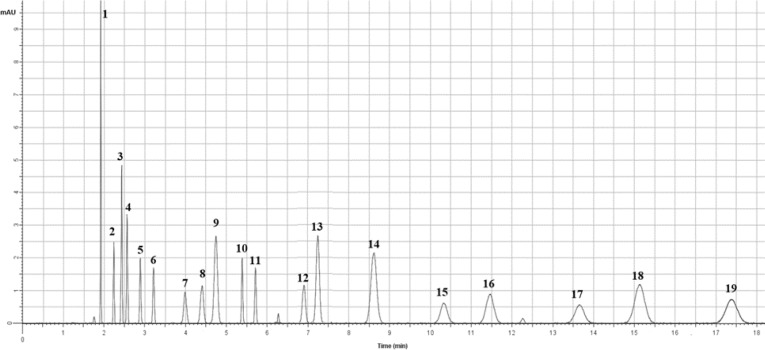
HPLC analysis of *Acanthus* *syriacus* EtOH extract: (1) protocatechuic acid, (2) p-hydroxybenzoic acid, (3) vanillic acid, (4) caffeic acid, (5) syringic acid, (6) p-coumaric acid, (7) sinapic acid, (8) Ferulic acid, (9) kromeic acid, (10) chlorogenic acid, (11) rutin, (12) luteolin-glucoside, (13) apigenin-glucoside, (14) rosmarinic acid, (15) quercetin, (16) kaempferol, (17) luteolin, (18) naringenin, and (19) apigenin.

The extract was then fractionated utilizing silica gel column-chromatography (75×15 cm). The column was developed by a gradient mobile phase: one bed volume (BV) of hexane/ethyl acetate (50:50, v/v), then one BV EtAc, then one-BV of EtAc/water/formic acid (46:46:8, v/v), then 2 BV of EtAc, formic acid, water and hexane (70:7.5:7.5:15, v/v/v/v), then 1 BV ethanol/water (50:50, v/v), and finally one BV 100% water. Fractions were collected every 2 min. and similar fractions were combined and concentrated under reduced pressure. In order to identify the most active compound (s), each fraction was evaluated in the same-way as the *A. syriacus* extract for its antinociceptive properties. Most fractions were identified by steeping method utilizing the RP-HPLC system and utilizing reference standards. Peak nine was (Figure 1) identified utilizing ^1^H and ^13^C NMR analysis.

**Sample preparation for** ^1^ **H and** ^13^ **C NMR investigation**

The NMR experiments were done using a Bruker 300 MHz spectrometer (Germany) equipped with an auto-sampler. NMR samples were prepared by dissolving the isolated compound(s) in deuterated methanol (MeOH-d4, Sigma-Aldrich). NMR spectra ^13^C-HSQC (Hetero-nuclear single quantum coherence), ^13^C-HMBC (Hetero-nuclear Multiple Bond coherence), ROESY NMR (Rotating-frame Overhauser Effect Spectroscopy) and COSY NMR (Correlation Spectroscopy) were obtained at 300 MHz, at 25°C. 

**Animals**

Male albino mice (22-30g) were accommodated for one week prior to the *in vivo *experiment. The animals had free-access to water and standard feeding pellets (except otherwise stated), and were kept under 12hr/12hr dark/light cycles. All experiments were done according to animal-care rules and regulations, and approved by BAU Institutional Review Board (2019A-0056-P-R-0297).

**Acute carrageenan-provoked inflammatory-pain**

In order to assess the acute carrageenan-induced inflammatory-pain, 100μL of 1% carrageenan-solution was intraplantarly injected into the mice left hind-paw (n=7/group). The positive control (ibuprofen 100mg/kg) was orally-administered 0.5hr prior to carrageenan injection as described previously (Gardmark et al., 1998; Salama et al., 2016). The vehicle control (VEH) animals were injected intraplantarly with 100μL vehicle only (saline). Then, 120 min post-carrageen-injection, behavioral-measurements were performed.

**Diabetes induction**

Diabetes was induced in mice by injecting 180mg/kg alloxan every other day for three days. The blood glucose level (BGL) was checked by pricking the animal tail and utilizing Accu-chek glucometers (Germany) (Jamalan et al., 2015 ▶; Khaneshi et al., 2013 ▶). The blood glucose levels (BGL) were measured acutely at hour 6 and subchronically for 8 days, and for 8 weeks. Sigma glucometers (Germany) were utilized to monitor BGL. The glycated-hemoglobin (HbA1c) level was monitored utilizing Analyticon HbA1c kits (Germany) for 8 weeks. Animals having BGL≥200 mg/dL and HbA1c > 8 were considered diabetic.

**Experimental protocol**

After confirmation of diabetes mellitus (DM) and NP after monitoring of basal nociceptive-reaction 8 weeks post-alloxan injection, mice were randomized to different groups (n=7/group), and scheduled to pre- and 8-week post-treatment monitoring, as follows:

- Test groups: The *A. syriacus* ethanol extract (AS) (50, 100, and 150mg/kg), FKCA isolated fraction (FKCA) (5, 10, and 20mg/kg), kromeic acid (KRA) (5, 10, and 20mg/kg) were given to alloxan-induced diabetic-mice, and treated every other day for 8 weeks, and monitored pre- and 8-week post-treatment.
- Positive controls: The following positive standards were used: ibuprofen 100mg/kg (Ib) in inflammatory pain analysis, glibenclamide (5mg/kg) (GB) in HbA1c and serum insulin experiments, tramadol 10 mg/kg (TRA) in diabetic neuropathy experiments, and metformin 25mg/kg (MTF) in the biochemical studies.
- Vehicle control (VEH or DIA+VEH): Group of alloxan-induced vehicle-treated diabetic control mice.
- Normal control (NORM): Group of normoglycemic non-treated normal mice.

**HbA1c and serum insulin levels**

The glycated-hemoglobin (HbA1c) level was carefully monitored prior to treatment and eight-week post-treatment utilizing Analyticon HbA1c analytical columns. Moreover, the serum-insulin levels were measured pre- and 8-week post--oral administration using a reversed phase HPLC method utilizing RP-C18 endcapped Lichrospher-column (Merck) at 40°C with a flow-rate of 1ml/min. The solvent for HPLC was composed of trifluoroacetic acid (0.1%) in double-distilled water (DDW) (A) and acetonitrile (ACN) (B). The gradient-elution-conditions at 214 nm were as follows: 0 min 70% (A), and then 5min 60% (A), as described before (Raafat et al., 2017a ▶; Raafat and Wael, 2018 ▶).

**Oxidative-stress analysis**

In order to evaluate the oxidative-stress, serum-catalase (CAT, kU/I) and reduced glutathione (GSH, µg/mg) were monitored pre- and eight-week post- oral administration (Ellman, 1959; Yasmineh et al., 1995). Utilizing a modified method, the lipid peroxidation (LPO) levels have been evaluated by thiobarbituric acid(TBA) experiment (Ohkawa et al., 1979). Concisely, pre- and 8-week after-treatment, TBA (0.8%) was added to serum (0.2mL), sodium lauryl sulfate (SLS, 8.1%), and dilute HAc in DDW (20%). Following 60 min of heating at 95°C and then cooling-down, the combination was extracted using methanol/isopropyl alcohol (1:15, v/v), and then the absorbance was measured by a JASCO-spectrophotometer (Japan) at 532 nm (Ohkawa et al., 1979 ▶).

**Nociceptive responses assessment**

Eight weeks after diabetes induction, animals were evaluated for diabetic-neuropathy success-rate (DNSR, significant losing sensory response to thermal-nociception below 10 sec (Sullivan et al., 2007). The DNSR was about 88% and the test compounds antinociceptive potentials were evaluated every other week for eight weeks.

**Thermal-nociceptive latency evaluation**

Animals with DNSR were involved in the thermal-hyperalgesia tail-flick and hot-plate latency experimentations (Micov et al., 2015). In brief, the animals were tested utilizing a hot-plate analgesia-meter (Ugo-Basile-Italy), or a tail-flick apparatus (Hugo-Sachs-Elektronik-Germany). The thermal-intensity was tuned to provide a baseline latency-time for the hot-plate test of 4–5 sec, and 1.5-2.5 sec for the tail-flick test for normal non-diabetic mice (NORM). A 10-sec cut-off time was adopted in thermal-nociceptive latency experiments to avoid tissue-damage.

**Mechanical-nociceptive latencies evaluation**

The tactile-allodynia, in mice with DNSR, was evaluated by monitoring the paw-withdrawal-thresholds (PWT) using Von-Frey-filaments (OptiHair) (Ohsawa et al., 2011 ▶). In brief, mice were separately put on a mesh floor in a bottom-up plastic cage. The force on the plantar-surface of the animal left hind-paw was gradually increased till it withdrew the paw. Here, 32 g cut-off force was adopted in the mechanical-nociceptive experiments for animal safety.

**Nitric oxide (NO) levels measurement**

NO level was determined by utilizing the Griess-reagent technique (Green et al., 1982 ▶). Both urinary (µM/L) and tissue (µM/mg protein) nitrite were measured.

**Statistical analysis**

Outcomes (mean ± SEM) were statistically assessed by one way ANOVA followed up by the Student–Newman–Keuls analysis utilizing OriginPro® statistics-software. Ap-value˂0.05 was regarded as statistically-significant.

## Results

**HPLC-PDA analysis:** ***A. syriacus*** **whole extract standardization**

HPLC-PDA analysis revealed the phenolic compounds present in the in *A. syriacus *ethanolic extract (Figure 1).The HPLC chromatogram showed 19 major peaks, from them 18 peaks were identified by steeping method utilizing standard curves. One compound (peak 9) was elucidated by ^1^H and ^13^C NMR as a novel quinic acid derivative and was named kromeic acid (Figure 1).The chemical structures and the percentages variation of the main compounds is presented in Table 1. The RP-HPLC major peaks were: (1) protocatechuic acid (3.47%), (2) p-hydroxybenzoic acid(6.47%), (3) vanillic acid (5.50%), (4) caffeic acid (3.60%), (5) syringic acid (3.12%), (6) p-coumaric acid (2.90%), (7) sinapic acid (3.41%), (8) ferulic acid (3.49%), (9) kromeic acid (6.10%), (10) chlorogenic acid (3.10%), (11) rutin (2.84%), (12) luteolin-glucoside (4.10%), (13) apigenin-glucoside (6.23%), (14) rosmarinic acid (8.43%), (15) quercetin (4.20%), (16) kaempferol(6.80%), (17) luteolin (3.80%), (18) naringenin (11.30%), and (19) apigenin (9.33%) (Figure1 and Table1). Quantitative tests showed that naringenin and apigenin were the most abundant phenolic compounds in the *A. Syriacus *ethanolic extract. Comparatively lower amounts of rutin, p-coumaric acid, and chlorogenic acid also remarkably distinguished *A. syriacus* sample (Figure 1). 

**Table1:** Phenolic components found in the RP-HPLC studied *A. syriacus* ethanolic extract.

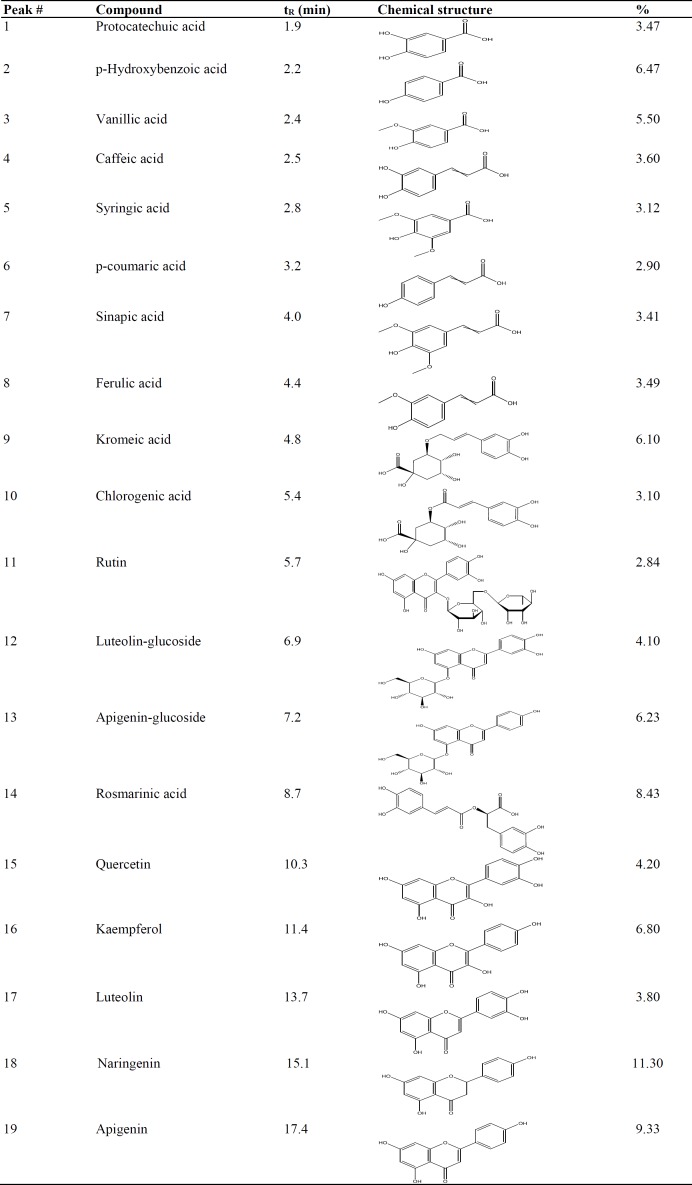

***A. syriacus*** **bio-guided isolation, RP-HPLC,** ^1^ **H and** ^13^ **C NMR structure elucidation and identification of the most active compounds**

**Figure 2:**
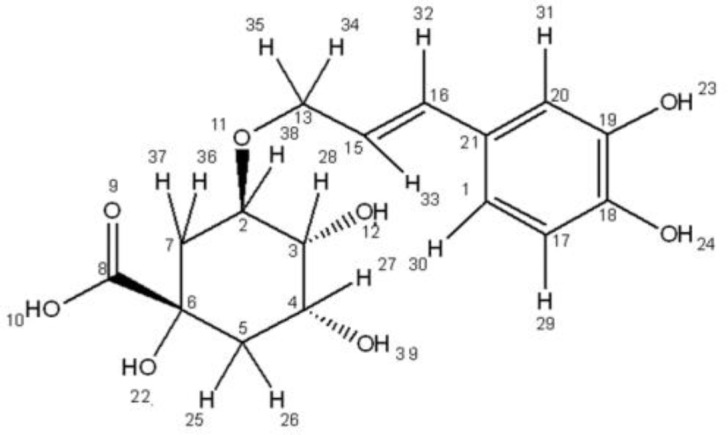
Kromeic acid (KRA) chemical structure

The most active fraction having antinociceptive potentials was isolated utilizing column-chromatography separation method. The RP-HPLC analysis, similar to the whole extract, was done for the most active fraction. Three compounds were recognized in this active fraction. Two compounds of the most active fraction were identified as ferulic acid and chlorogenic acid by steeping method utilizing reference standards. The third compound was isolated from the mixture by subjecting it to second silica-gel column-chromatography and was developed using dichloromethane and mixtures of dichloromethane and methanol with increasing polarities. Similar fractions were combined and concentrated. The third compound was further purified by semi-preparative HPLC. Compound 3 was obtained as an amorphous yellowish solid and identified by m/z: 340.1200 using Nano-ESI-MS in the positive mode, elemental analysis: C, 56.5%; H; 5.9%; O, 37.6%, and chemical formula C_16_H_20_O_8_. The ^1^H Spectral data (Table 2 and Figure 2) exhibited 2.14, dd (J=14.50, 2.79 Hz), 2.13, dd (J=14.50, 2.79 Hz), 2.34, dd (J=14.25, 2.79 Hz), 2.33, dd (J=14.25, 2.79 Hz), 3.18, t (J=2.79 Hz), 3.3, dt (J=10.26, 2.79 Hz), 3.40, q (J=2.79 Hz), 3.81, d (J=7.30 Hz), 3.81, d (J=7.30 Hz), 5.60, s, 5.90, s, 6.25, dt (J=17.56, 7.30 Hz), 6.50, d (J=17.56 Hz), 7.06, dd (J=8.56, 1.90 Hz), 7.41, dd (J=8.56, 1.90 Hz), 7.42, dd (J=8.60, 1.90 Hz), 9.48, s, 9.48, s. The three dd [7.06, dd (J=8.56, 1.90 Hz), 7.41, dd (J=8.56, 1.90 Hz), 7.42, dd (J=8.60, 1.90 Hz)] belonged to the aromatic protons. The olefinic protons were observed at 6. 25, dt (J=17.56, 7.30 Hz), and 6.50 d (J=17.56 Hz). The quinic acid protons appeared at 2.14, dd (J=14.50, 2.79 Hz), 2.13, dd (J=14.50, 2.79 Hz), 2.34, dd (J=14.25, 2.79 Hz), 2.33, dd (J=14.25, 2.79 Hz), 3.18, t (J=2.79 Hz), 3.3, dt (J=10.26, 2.79 Hz), 3.40, q (J=2.79 Hz). The methylene group protons appeared as two duplets at δ 3.81. The main proton-proton neighboring interactions of the aromatic ring were detected between H-29 and H-30 in the COSY spectrum. The ^13^C NMR chemical shifts (Table 2) at δ114.8, δ 116.1, δ 123.0, δ 130.7, δ 132.3, δ 146.5, and δ 146.5 were assigned to the aromatic carbons. Comparison of the ^13^C NMR with HMQC and DEPT spectra revealed C_13_ methylene group. The olefinic group carbon appeared at δ123.1 and δ132.3. The quinic acid carbons appeared at δ38.1, δ38.3, δ70.3, δ71.8, δ73.5, andδ74.5, with the carboxylic acid carbon observed at δ 180.4. TheC_13_ signal of the methylene group at δ66.6 exhibited a clear correlation with the olefinic proton at δ 123.1 in the HMQC spectrum. The HMBC spectrum of the compound expounded a signal between the C_2_of the quinic acid and the H_35_of the methylene group (Table 2 and Figure 2). Therefore, the quinic acid derivative (compound 3) was named kromeic acid (KRA), and the most active fraction in *A. syriacus* (AS) was named FKCA due to its content of ferulic acid (27.5%), kromeic acid (48.1%), and chlorogenic acid (24.4%).

**Table 2:** Kromeic acid (KRA) ^1^H -NMR and ^13^C- NMR data.

**Position** *	**δC**	**Position** *	**δH, m, (** ***J*** ** in Hz)**
**1**	123.0	22	5.90, s
**2**	74.5	23	9.48, s
**3**	73.5	24	9.48, s
**4**	70.3	25	2.33, dd (*J*=14.25, 2.79Hz)
**5**	38.1	26	2.34, dd (*J*=14.25, 2.79Hz)
**6**	71.8	27	3.3, dt (*J*=10.26, 2.79Hz)
**7**	38.3	28	3.18, t (*J*=2.79Hz)
**8**	180.4	29	7.42, dd (*J*=8.60, 1.90Hz)
**9**	__	30	7.41, dd (*J*=8.56, 1.90Hz)
**10**	__	31	7.06, dd (*J*=8.56, 1.90 Hz)
**11**	__	32	6.50, d (*J*=17.56Hz)
**12**	__	33	6.25, dt (*J*=17.56, 7.30Hz)
**13**	66.6	34	3.81, d (*J*=7.30Hz)
**14**	__	35	3.81, d (*J*=7.30Hz)
**15**	123.1	36	2.13, dd (*J*=14.50, 2.79Hz)
**16**	132.3	37	2.14, dd (*J*=14.50, 2.79Hz)
**17**	116.1	38	3.40, q (*J*=2.79Hz)
**18**	146.5	39	5.60, s
**19**	146.5		
**20**	114.8		
**21**	130.7		

* As shown in Figure2.

**AS, FKCA and KRA potentials against inflammatory-pain**

**Figure 3:**
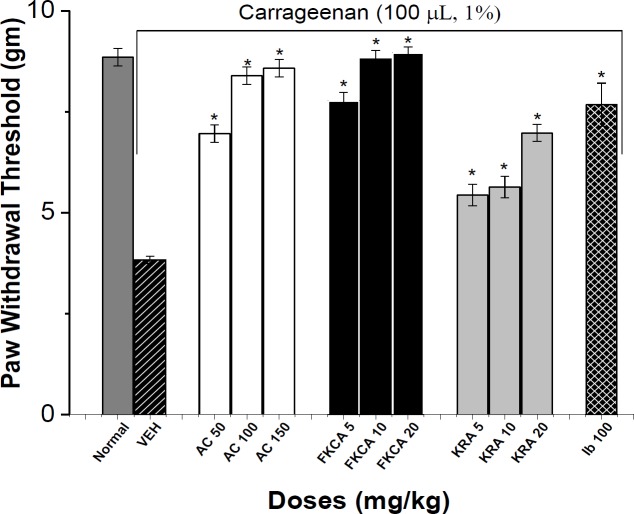
Inflammatory pain analysis. Carrageenan (100µL, 1%) was injected intraplantarly 2hr prior to the pain threshold assessment by the paw pressure test. Oral administration (gavages) of *Acanthus syriacus *(AC)*, *FKCA isolated fraction(FKCA), and kromeic acid (KRA) 0.5hr before the test. Oral Ibuprofen 100mg/kg (Ib 100) was used as positive-control.“Normal” designates normal-mice. "*" means p<0.05compared to vehicle-control (VEH) (n=7/group).

To evaluate AS, FKCA, and KRA anti-inflammatory activities, inflammation acute-phase was induced in a mouse model of paw-edema by carrageenan. Two hours after intraplantar carrageenan injecting to the animals, a significant (p<0.05) mechanical hypersensitivity was induced (n=7/group) (Figure 3). The PWT was reduced from 8.84±0.19g in normal-mice (Normal) to 3.84±0.06g in the vehicle-control group (VEH). AS in a dose-dependent manner significantly ameliorated the carrageenan-induced edema (n=7/group). As compared to VEH, AS (50, 100 and 150mg/kg) elevated PWT (i.e. reversed the mechanical-hypersensitivity) with0.79, 1.16 and 1.23 folds, respectively. In a dose-dependent manner, the most active fraction (i.e. FKCA) and the isolated KRA significantly ameliorated the carrageenan-induced edema (n=7/group). Compared to VEH, FKCA (5, 10 and 20mg/kg) elevated PWT by 1.00, 1.26 and 1.31 folds, respectively, whereas KRA (5, 10 and 20mg/kg) elevated PWT by 0.40, 0.45 and 0.82 folds, respectively (Figure 3).The efficiency of AS, FKCA, and KRA on reversing mechanical-hypersensitivity, proposes their capabilities to ameliorate acute inflammatory-pain.

**AS, FKCA and KRA potentials against BGL, HbA1c, and serum insulin**

**Figure 4:**
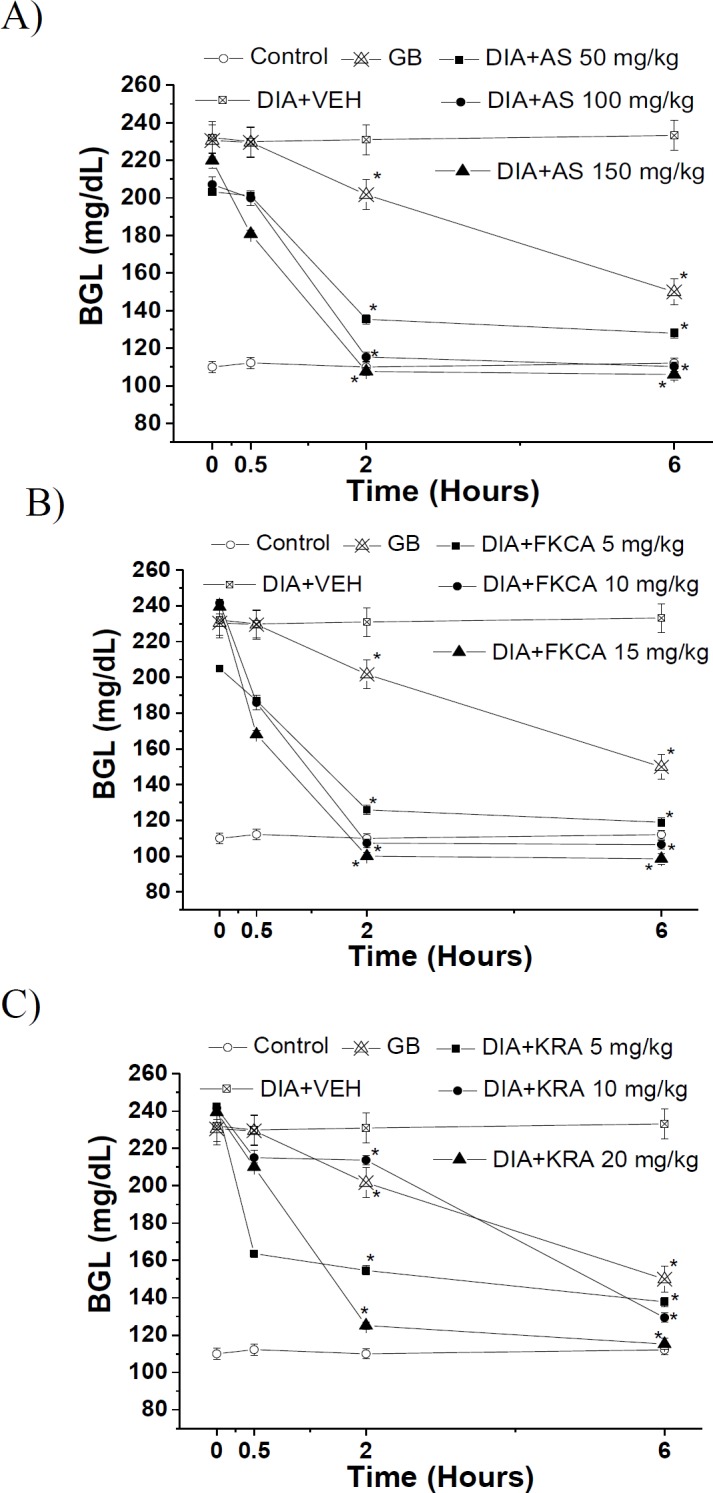
The acute effect of (A) *Acanthus syriacus *(AC), (B) FKCA isolated fraction (FKCA), and (C) kromeic acid (KRA)on blood glucose levels, utilizing glibenclamide 5mg/kg (GB) as positive control. “Control” designates normal non-diabetic mice. “*” designates significant differencesatp˂0.05when compared to vehicle-treated diabetic control (DIA+VEH) (n=7/group).

In the acute experiments, AS (50, 100 and 150mg/kg) reduced BGL by 45.4, 52.7 and 54.5%, 6hr post-administration, respectively, as compared to vehicle-treated diabetic control mice (DIA+VEH) (Figure4A). When compared to DIA+VEH group, FKCA (5, 10 and 20mg/kg) reduced BGL by 49.1, 54.4 and 57.6%, 6hr post-administration, respectively (Figure 4B).KRA (5, 10 and 20mg/kg) decreased BGL by 40.6, 44.1 and 50.9%, 6hr post-administration, respectively. However, glibenclamide 5mg/kg (GB), produced 35.9% reduction in BGL (Figure4C).

**Figure 5:**
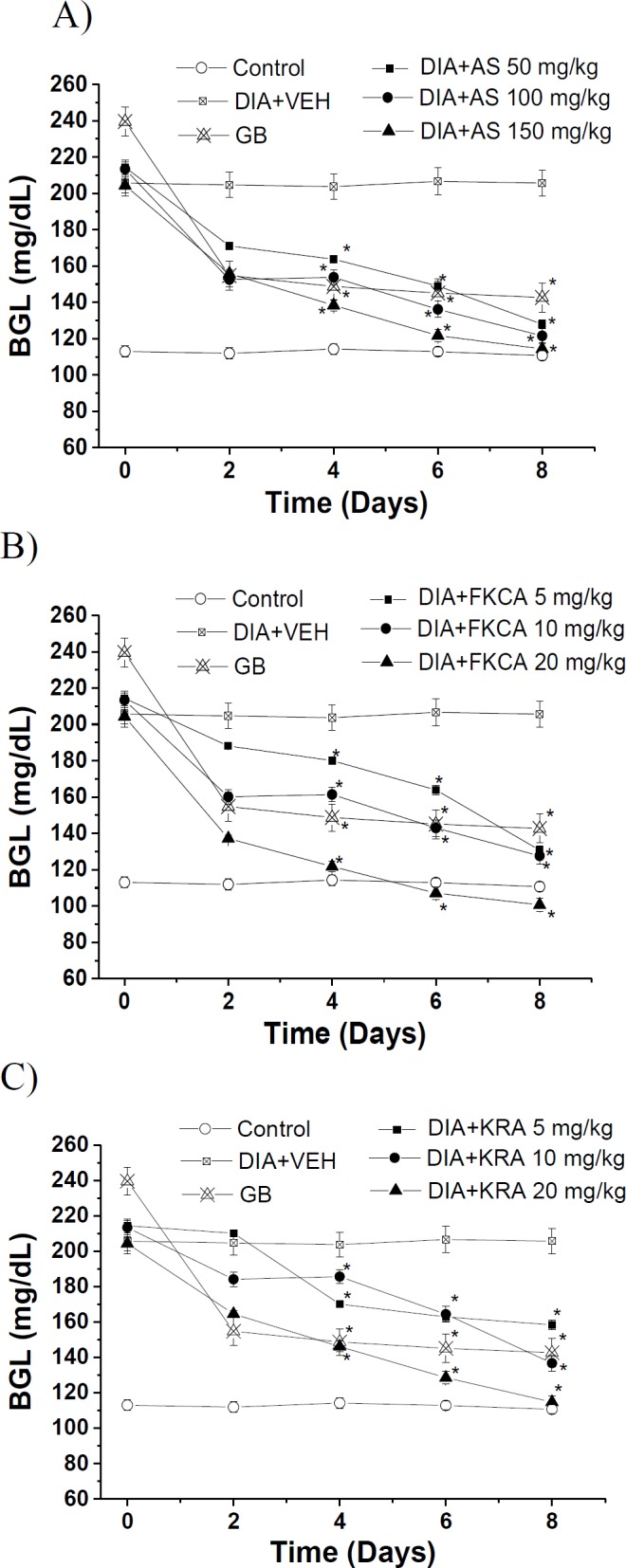
The subchronic effect of (A) *Acanthus syriacus* (AC), (B) FKCA isolated fraction (FKCA), and (C) kromeic acid (KRA)on blood glucose levels, utilizing glibenclamide 5mg/kg (GB) as the positive control. “Control” designates normal non-diabetic mice. “*” designates significant differences atp˂0.05when compared to vehicle- treated diabetic control (DIA+VEH) (n=7/group).

Moreover, subchronically, AS (50, 100 and 150 mg/kg) declined BGL by 37.9, 41.1 and 44.2%, 8-day post-administration, respectively, when compared to DIA control (Figure5A). Compared to DIA+VEH, FKCA (5, 10 and 20 mg/kg) declined BGL by 36.7, 37.7 and 51.1%, 8-day post-administration, respectively (Figure 5B); nevertheless, KRA (5, 10 and 20mg/kg) reduced BGL by 22.9, 33.1 and 43.9%, 8-day post-administration, respectively. GB causeda30.5% reduction in BGL (Figure 5C).

**Figure 6:**
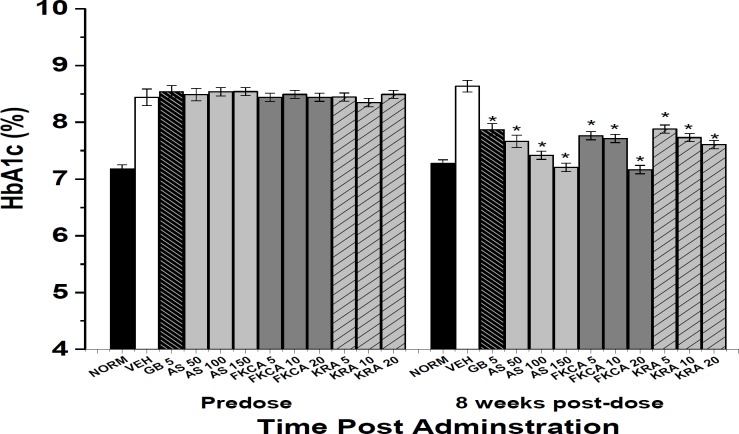
The effect of *Acanthus syriacus* (AC*),* FKCA isolated fraction (FKCA), and kromeic acid (KRA) (mg/kg) on HbA1c levels before (pre-dose) and 8 weeks after administration (8-week post-dose), utilizing glibenclamide 5mg/kg (GB) as positive control. “NORM” designates normal non-diabetic mice. “*” designates significant differences (p˂0.05) as compared to vehicle-treated diabetic control (VEH), (n=7/group).

Increasing doses of AS, FKCA, and KRA significantly and dose-dependently decreased HbA1c level 8- week post-administration (Figure 6). Compared to VEH, AS (50, 100 and 150mg/kg) significantly reduced HbA1c by 10.8, 13.8 and 16.2%, respectively (Figure6). FKCA (5, 10 and 20mg/kg) also reduced the HbA1c levels 8-week post-administration by 9.9, 10.2 and 16.6%, respectively. KRA (5, 10 and 20mg/kg) ameliorated HbA1c by 8.1, 10.1 and 11.5%, respectively. However, GB caused a 8.4% reduction in the level of HbA1c (Figure 6).

**Figure 7:**
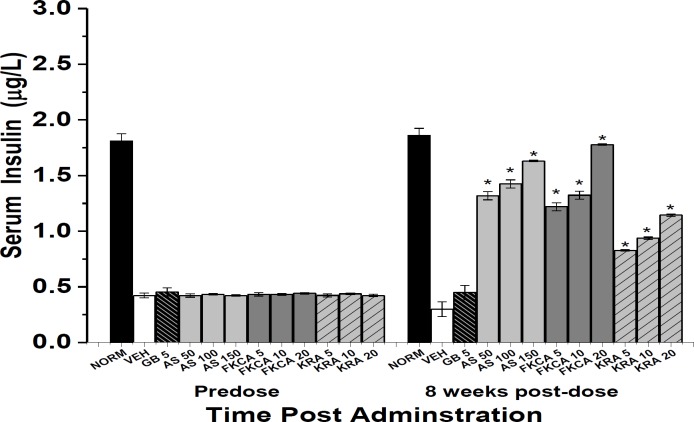
The effect of *Acanthus syriacus *(AC), FKCA isolated fraction (FKCA), and kromeic acid (KRA) (mg/kg) on serum insulin levels before (predose) and 8 weeks after administration (8-week post-dose), utilizing glibenclamide 5mg/kg (GB). “NORM” designates normal non-diabetic mice. “*” designates significant differences (p˂0.05) ascompared to vehicle-treated diabetic control (VEH), (n=7/group).

To better evaluate AS, FKCA, and KRA antidiabetic mechanism, serum insulin levels were monitored. In a dose-dependent manner and after 8-weekoral administrations, AS, FKCA, and KRA significantly raised serum insulin level (SIL) (Figure 7). AS (50, 100 and 150mg/kg) significantly raised SIL by 3.4, 3.8 and 4.4 folds, respectively, compared to VEH (Figure 7). As compared to VEH, FKCA (5, 10 and 20mg/kg) raised the SIL by 3.1, 3.4 and 4.9 folds, respectively, likewise, KRA (5, 10 and 20mg/kg) raised the SIL by 1.8, 2.1 and 2.8 folds, 8-week post-administration, respectively. In contrast, GB did not raise SIL significantly (Figure 7).

The potencies of AS, FKCA, and KRA on ameliorating BGL and controlling HbA1c levels, propose their potentials to control diabetes and NP.

**Figure 8:**
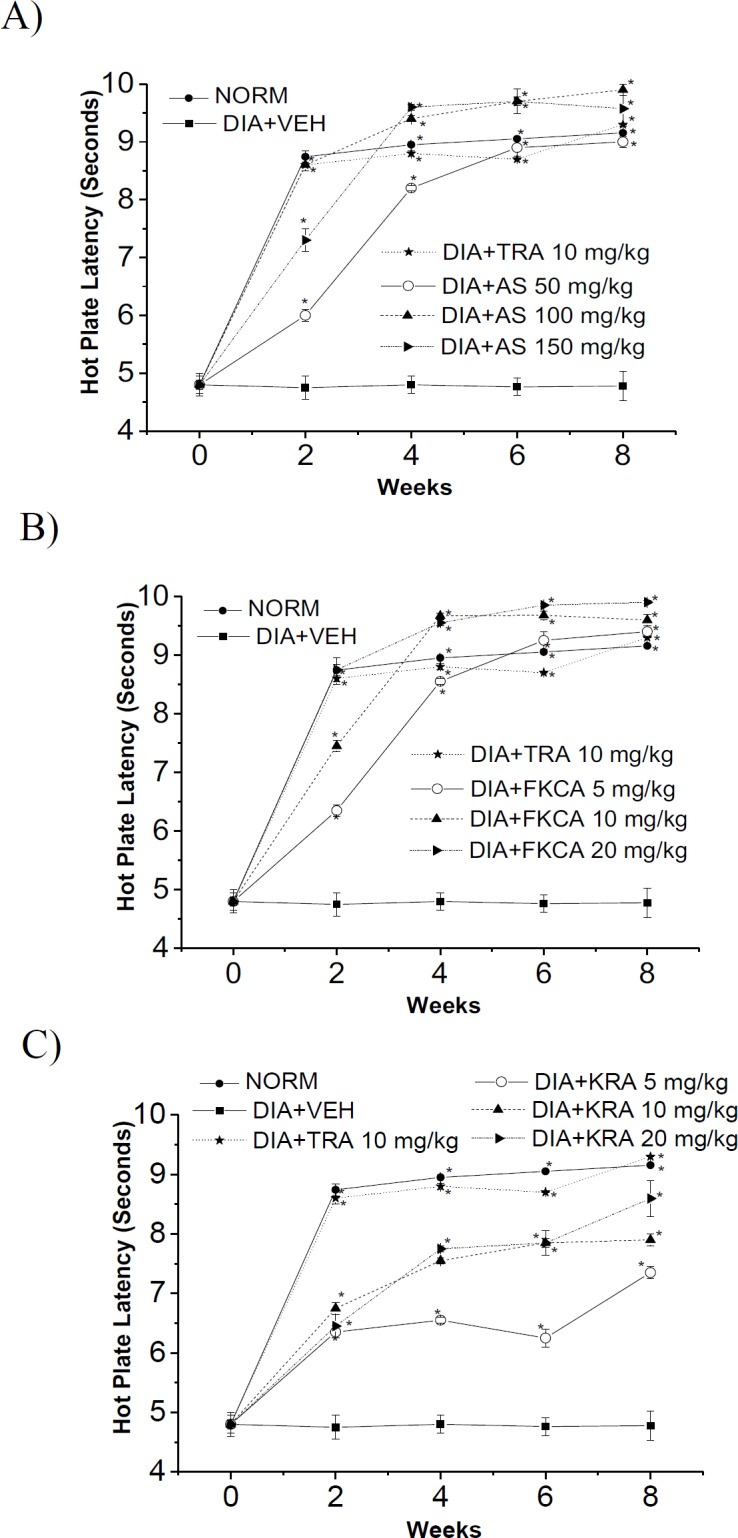
The effect of (A) *Acanthus syriacus*(AC),(B) FKCA isolated fraction (FKCA), and (C) kromeic acid (KRA) against hyperalgesia on the hot-plate latency in alloxan-treated mice with tramadol (TRA 10mg/kg) as a positive control. “NORM” designates normal non-diabetic mice. "*" shows significant differences atp<0.05, compared tovehicle diabetic-control (DIA+VEH) (n=7).

**Serum catalase (CAT), reduced glutathione (GSH), and lipid peroxide (LPO) levels**

CAT, GSH, and LPO levels were measured at pre- and 8-weekpost-treatments, and compared to VEH. AS (50, 100 and 150mg/kg) significantly elevated CAT level by 1.32, 1.50, and 1.57 folds, increased GSH level by 87.5, 88.7, and 95.3%, , and reduced LPO level by 92.3, 92.1 and 92.6%, respectively (Table 3). FKCA (5, 10 and 20 mg/kg) raised the CAT level by 1.28, 1.45 and 1.58 folds, increased GSH level by 84.8, 86.3, and 98.2%, and reduced LPO level by 92.1, 91.7 and 92.8%, respectively, KRA (5, 10 and 20mg/kg) raised the CAT level by 0.80, 0.94, and 1.01 folds, increased GSH level by 59.5, 64.6, and 65.1%, and reduced LPO level by 89.4, 90.0 and 90.5%, respectively (Table 3).

**Nociceptive responses assessment**

**Figure 9:**
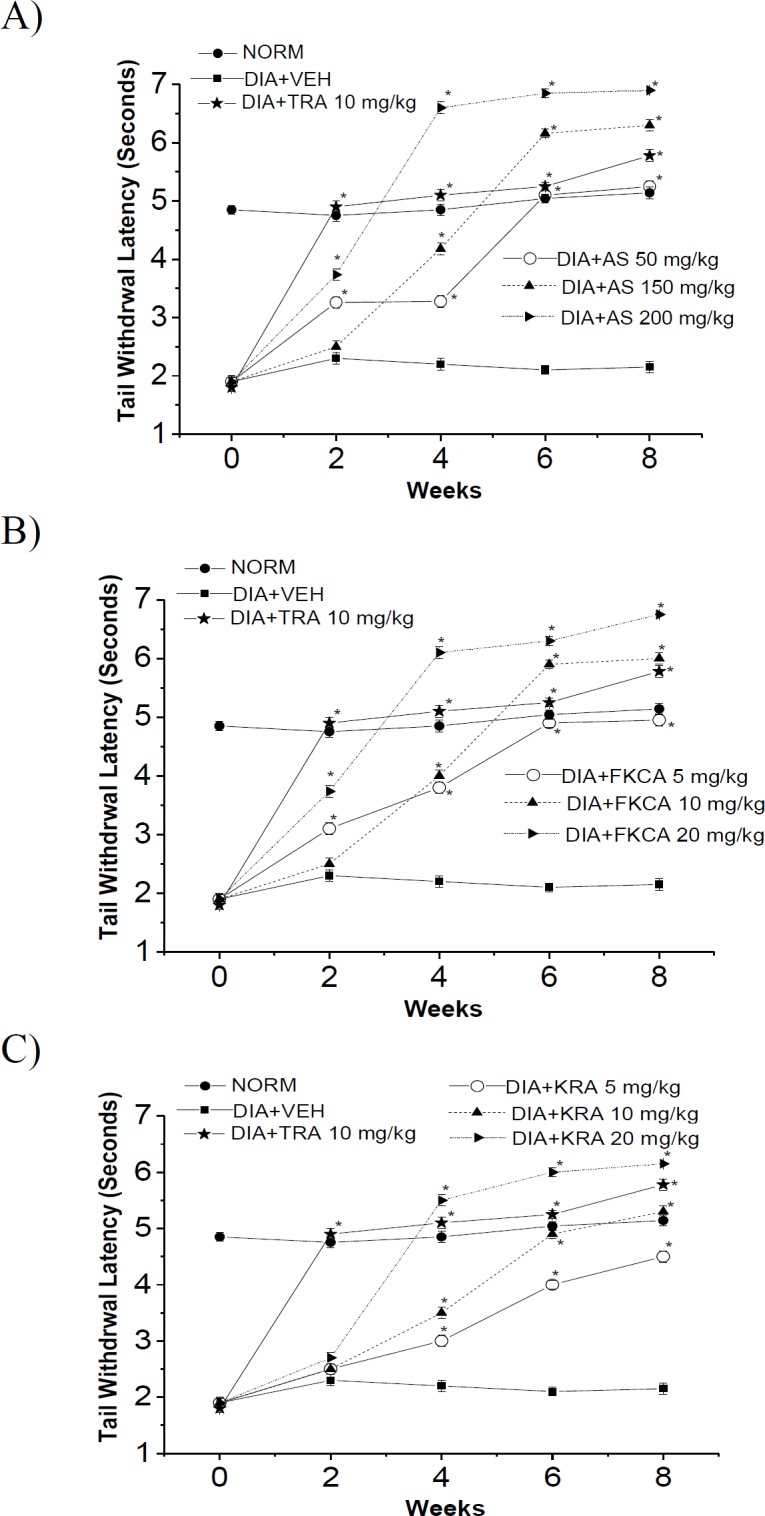
The effect of (A) *Acanthus syriacus* (AC), (B) FKCA isolated fraction (FKCA), and (C) kromeic acid (KRA) against hyperalgesia in terms of tail-flick latency in alloxan-treated mice treated with tramadol (TRA 10mg/kg) as a positive control. “NORM” designates normal non-diabetic mice. "*" shows significant differences at p<0.05compared to vehicle diabetic-control (DIA+VEH), (n=7).

**Figure 10:**
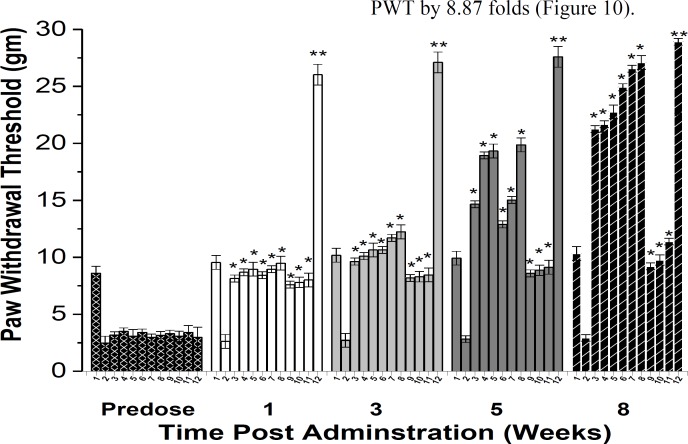
The effect of *Acanthus syriacus* (AC), FKCA isolated fraction (FKCA), kromeic acid (KRA), and tramadol (TRA) 10 mg/kg, on tactile allodynia in a neuropathic model; 1: (NORM) normal non-diabetic untreated mice, 2: VEH, 3: AS 50 mg/kg, 4: AS 100 mg/kg, 5: AS 150 mg/kg, 6: FKCA 5 mg/kg, 7: FKCA 10 mg/kg, 8: FKCA 20 mg/kg, 9: KRA 5 mg/kg, 10: KRA 10 mg/kg, 11: KRA 20 mg/kg, 12: TRA 10 mg/kg (n=7 animals/ group). *p≤0.05 and **p≤0.01 show significant differences compared to vehicle-treated animals (VEH).

Thermal and tactile neurological-functions were evaluated every other week for 8 weeks post-treatment exploiting hot-plate latency (HPL), tail-flick latency (TFL), and von Frey filament paw-withdrawal thresholds (PWT) methods (Figures 8, 9 and 10).

**Thermal-pain responses**

After 8 weeks of oral administration of different treatments, as compared to VEH, the orally-administrated AS 50, 100 and 150mg/kg provoked a significant rise in the thermal-stimuli reaction-time by 0.88, 1.07 and 1.00 folds for HPL, and by 1.44, 1.93 and 2.21 folds for TFL, respectively (Figures8A and 9A). Also, FKCA administration at doses of 5, 10 and 20mg/kg, raised HPL by 0.96, 1.01 and 1.07 folds, and elevated TFL by 1.30, 1.79, and 2.16 folds, respectively (Figures 8B and 9B). Moreover, KRA oral administration at doses of 5, 10 and 20mg/kg raised HPL by 0.53, 0.65, and 0.80 folds, and raised TFL by 1.10, 1.47 and 1.86 folds, respectively (Figures 8C and 9C). These outcomes were evaluated against tramadol 10mg/kg (TRA), a positive control, which increased HPL by 0.94 folds, and TFL by 1.69 folds (Figures 8 and 9).

The efficiency of AS, FKCA, and KRA on amelioration of thermal hyperalgesia, proposes their antinociceptive properties against hyperalgesic-pain. 

**Tactile-nociceptive responses**

The tactile allodynia was evaluated by assessing PWT exploiting Von-Frey filaments after 8-week oral administration of different treatments and compared to VEH group (Figure 10). AS at doses of 50, 100 and 150mg/kg induced a significant rise in PWT by 6.25, 6.40 and 6.77 folds, respectively (Figure 10).FKCA at doses of 5, 10 and 20mg/kg elevated PWT by 7.51, 8.06, and 8.25folds, respectively (Figure10).Also, KRA 5, 10 and 20 mg/kg raised PWT by 2.14, 2.33 and 2.88 folds, respectively (Figure 10). TRA elevated PWT by 8.87 folds (Figure 10).

**AS, FKCA and KRA potentials against urinary and tissue nitrite level**

One of the possible anti-neuropathic pain mechanisms of action of a given compound is mediated through it potential to reduce nitrite level (Taliyan and Sharma, 2012). Thus, nitrite levels in the urine and heart left ventricle tissues were monitored pre- (control group) and 8-weeks post-treatment (test group), in order to explore the test compounds’ antinociceptive mechanism. The vehicle-treated neuropathic animals significantly raised urinary and tissue nitrite concentration compared to normal control (Table4). Compared to vehicle control, the AS (50, 100, and 150mg/kg) reduced urinary nitrite level (UNL) by 23.6, 53.3, 58.1%, respectively, and decreased tissue nitrite level (TNL) by 14.5, 48.9, 58.8%, 8-week post-treatment (Table 4). Eight-week oral administration of FKCA (5, 10, and 20mg/kg) declined UNL by 22.0, 50.8, 59.3%, respectively, and reduced TNL by 20.8, 40.3, and 60.6%, whereas KRA (5, 10, 20mg/kg) declined UNL by 17.4, 38.7, and 48.6%, respectively, and decreased TNL by 16.9, 32.3, and 46.6%, respectively, compared to vehicle control (Table 4). The positive control, MTF, had no significant effect on UTL or TNL (Table 4).

**Table 3:** *In-vivo* assessment of the antioxidant activities of *Acanthus syriacus* (AC), FKCA isolated fraction (FKCA), and kromeic acid (KRA) on serum levels of CAT, reduced GSH, and alterations in TBARS (n=7/group; data presented as Mean ± SEM).

**Group**	**Dose (mg/kg)**	**Catalase level (kU/I)**		**GSH (µg/mg)**		**TBARS Level (nM/100g)**	
		Predose	8 weeks	Predose	8 weeks	Predose	8 weeks
**Normal control**	__	32.03±1.40	31.05±1.58	60.30±1.09	60.70±1.08	0.87±0.01	0.84±0.01
**Vehicle control**	__	22.16±1.00	16.70±1.13	55.53±1.60	39.50±1.50	0.98±0.02	5.30±0.01
**MTF** ^a^	25	21.93±1.13	22.98±1.18	55.10±1.40	53.90±1.30	1.02±0.01	1.35±0.02
**AS** ^a^	50	22.54±1.03	38.87±1.25 *	55.43±1.50	74.07±1.00 *	0.91±0.01	0.41±0.01*
**AS** ^a^	100	22.88±1.21	41.85±1.30 *	54.56±1.40	74.52±1.10 *	0.93±0.02	0.42±0.02 *
**AS** ^a^	150	22.74±2.25	42.97±1.37 *	56.48±1.10	77.16±1.20 *	0.90±0.02	0.39±0.04 *
**FKCA** ^a^	5	23.10±1.46	38.10±1.05 *	56.39±1.40	73.01±1.50 *	0.90±0.01	0.42±0.02 *
**FKCA** ^a^	10	22.10±1.05	40.95±1.60 *	57.96±1.10	73.60±1.10 *	0.94±0.02	0.44±0.02 *
**FKCA** ^a^	20	23.41±1.60	43.05±1.51 *	57.44±1.20	78.30±1.20 *	0.89±0.03	0.38±0.01 *
**KRA** ^a^	5	22.29±1.38	30.11±1.35 *	56.04±1.10	63.02±1.10 *	1.06±0.01	0.56±0.01 *
**KRA** ^a^	10	22.17±1.03	32.32±1.04 *	57.18±1.30	65.00±1.00 *	0.95±0.02	0.53±0.01 *
**KRA** ^a^	20	22.70±1.68	33.60±1.05 *	55.61±1.60	65.20±1.40 *	0.90±0.01	0.50±0.02 *

SEM.: mean standard error

* p<0.05 indicates significant differences from the vehicle control animals.

a Compared to vehicle control.

**Table 4:** Effect of interventions on urinary and tissue nitrite level.

**Group**	**Dose (mg/kg)**	**Urinary nitrite level (µM/L)**		**Tissue nitrite level (µM/mg protein)**	
		Predose	8 weeks	Predose	8 weeks
**Normal control**	__	16.04±0.90	16.20±1.40	3.68±0.94	3.90±0.96
**Vehicle control**	__	37.97±1.94	41.76±1.92	11.48±0.50	12.63±0.60
**MTF** ^a^	25	36.65±1.01	34.65±2.10	10.10±1.22	9.02±1.00
**AS** ^a^	50	35.80±0.94	31.89±1.00 *	11.53±0.39	10.80±0.60*
**AS** ^a^	100	35.88±0.98	19.50±1.10 *	12.01±0.45	6.45±0.50 *
**AS** ^a^	150	36.01±1.09	17.47±1.07 *	11.56±0.6	5.20±0.50 *
**FKCA** ^a^	5	35.78±1.16	32.56±0.95 *	11.25±0.50	10.00±0.40 *
**FKCA** ^a^	10	36.02±0.94	20.55±1.30 *	10.98±0.40	7.54±0.45 *
**FKCA** ^a^	20	37.10±1.30	16.98±1.21 *	11.24±0.42	4.98±0.65 *
**KRA** ^a^	5	36.22±1.08	34.50±1.05 *	11.35±0.43	10.50±0.42 *
**KRA** ^a^	10	36.55±1.05	25.60±1.03 *	11.55±0.47	8.55±0.45 *
**KRA** ^a^	20	36.33±1.38	21.45±0.90 *	11.89±0.39	6.75±0.44 *

SEM.: mean standard error

* p<0.05 indicates significant differences from the vehicle control animals.

a Compared to vehicle control.

## Discussion

The phytochemical investigation, the bio-guided fractionation, and isolation schemes conducted in this study utilizing various chromatographic, NMR, and biological inflammatory and NP models, showed that *A. syriacus* most active fraction was the isolated FKCA fraction. Chromatographic methods identified ferulic and chlorogenic acids as two of the three components of FKCA by. The third compound was a quinic acid derivative elucidated by various analytical methods especially, ^1^H and ^13^C NMR method. The quinic acid derivative (compound 3) was named kromeic acid (KRA), and the most active fraction in *A. syriacus *(AS) was FKCA due to its content of Ferulic acid (27.5%), kromeic acid (48.1%), and chlorogenic acid (24.4%). 

Moreover, the significant efficiency of the tested compounds with respect to raising SIL, implies that the insulin-secretagogue activity is amongst diabetes controlling mechanisms of AS, FKCA, and KRA. These results are similar to previous reports indicating *Prunus cerasus *and *Anemone coronaria *secretagogue potential as one of their anti-diabetic mechanisms of action (Raafat and El-Lakany, 2018 ▶; Saleh et al., 2017 ▶). 

The antioxidant potentials of AS, FKCA, and KRA on elevating CAT and GSH, and reducing LPO levels, propose their potentials in ameliorating painful-neuropathy. Earlier studies on compounds with similar antioxidant potentials, also found antinociceptive properties (Muthuraman et al., 2008 ▶; Nishiyama and Ogawa, 2005 ▶).

AS, FKCA, and KRA ameliorating potentials on mechanical-nociceptive propose their antinociceptive activities against allodynic-pain. These results are aligned with earlier studies that suggested a neuroprotective effect for other natural-compounds, like Curcuma sesquiterpenes and *Gingko biloba *polyphenolics, against neurotoxicity (Hibatallah et al., 1999 ▶; Raafat and Omar, 2016 ▶; Shi et al., 2010 ▶).

Also, AS, FKCA, and KRA protective effects against NO-induced neuronal toxicity might be one of the test compounds’ possible antinociceptive mechanisms as described before for *G. biloba* extract and other natural compounds (Green et al., 1982 ▶; Taliyan and Sharma, 2012 ▶).

AS, FCKA, and KRA showed significant anti-inflammatory activities. Compared to TRA, highest doses of AS, FKCA, and KRA showed higher antinociceptive potentials in controlling allodynic and thermal-hyperalgesic NP. AS and FCKA showed relatively equipotent antinociceptive effects with a predominant ameliorating effect of FCKA at its highest dose (20mg/kg). FKCA had more marked antinociceptive effects than KRA suggesting additive effects of FKCA components strengthening its anti-neuropathic potentials. The insulin-secretagogue, anti-inflammatory, anti-oxidative-stress, and protective effects against NO-induced neuronal toxicity potentials may be amongst AS, FKCA and KRA mechanisms through which they alleviated neuropathic-pain.

After conducting clinical-trials, *A. syriacus, *FKCA, and KRA might be used as a novel complementary approach for alleviation of a variety of painful-syndromes.
